# B Cell Activating Factor (BAFF) Is Required for the Development of Intra-Renal Tertiary Lymphoid Organs in Experimental Kidney Transplantation in Rats

**DOI:** 10.3390/ijms21218045

**Published:** 2020-10-28

**Authors:** Louisa Steines, Helen Poth, Marlene Herrmann, Antonia Schuster, Bernhard Banas, Tobias Bergler

**Affiliations:** Department of Nephrology, University Hospital Regensburg, 93042 Regensburg, Germany; helen_reinfrank@web.de (H.P.); marlene-herrmann@gmx.net (M.H.); antonia-margarete.schuster@ukr.de (A.S.); bernhard.banas@ukr.de (B.B.); tobias.bergler@ukr.de (T.B.)

**Keywords:** tertiary lymphoid organs, kidney transplantation, B cells, BAFF

## Abstract

Intra-renal tertiary lymphoid organs (TLOs) are associated with worsened outcome in kidney transplantation (Ktx). We used an anti-BAFF (B cell activating factor) intervention to investigate whether BAFF is required for TLO formation in a full MHC-mismatch Ktx model in rats. Rats received either therapeutic immunosuppression (no rejection, NR) or subtherapeutic immunosuppression (chronic rejection, CR) and were sacrificed on d56. One group additionally received an anti-BAFF antibody (CR + AB). Intra-renal T (CD3^+^) and B (CD20^+^) cells, their proliferation (Ki67^+^), and IgG^+^ plasma cells were analyzed by immunofluorescence microscopy. Formation of T and B cell zones and TLOs was assessed. Intra-renal expression of TLO-promoting factors, molecules of T:B crosstalk, and B cell differentiation was analyzed by qPCR. Intra-renal B and T cell zones and TLOs were detected in CR and were associated with elevated intra-renal mRNA expression of TLO-promoting factors, including CXCL13, CCL19, lymphotoxin-β, and BAFF. Intra-renal plasma cells were also elevated in CR. Anti-BAFF treatment significantly decreased intra-renal B cell zones and TLO, as well as intra-renal B cell-derived TLO-promoting factors and B cell differentiation markers. We conclude that BAFF-dependent intra-renal B cells promote TLO formation and advance local adaptive alloimmune responses in chronic rejection.

## 1. Introduction

Kidney transplantation is the best available treatment for many patients with end-stage kidney failure; however, kidney allograft survival is limited, with a 10-year graft survival rate of only 56% [1]. A shortage of organs for donation is therefore exacerbated by premature allograft failure. On top of this, patients with multiple consecutive transplants have a higher risk of graft failure due to rejection [2]. Improving allograft survival is therefore an important aim in renal and transplantation medicine.

Apart from antibody-mediated rejection (ABMR), which is a major cause of allograft failure [3], kidney allograft inflammation is an important predictor of reduced allograft survival [4]. Subclinical inflammation is associated with poorer outcomes [4,5] and may precede irreversible interstitial fibrosis [6]. Due to its prognostic relevance, inflammation in areas of fibrosis has recently been incorporated into Banff rejection grading as an independent score (“iIFTA”, inflammation with interstitial fibrosis and tubular atrophy). The role of B cells in allograft inflammation has not been fully clarified. Intra-graft B cell clusters have been observed in animal and human transplant studies, and have been related to poorer outcomes [7,8]. Furthermore, overproportional amounts of B cells were found in allografts with subclinical rejection and fibrotic changes compared to grafts with subclinical rejection without fibrosis [9], suggesting a role of B cells in chronic rejection.

B cells can form organized follicular structures together with T cells and other immune cells [10,11]. Within these structures, termed tertiary lymphoid organs (TLOs), B cells can affect local immune activation [12]. TLOs resemble lymphoid follicles within secondary lymphoid organs and occur at sites of chronic inflammation. They have been detected in systemic lupus erythematodes (SLE), rheumatoid arthritis, multiple sclerosis, atherosclerosis, as well as heart and kidney transplantation, as reviewed by Pitzalis [13]. The formation of TLO is driven by local expression of factors required for formation of secondary lymphoid tissue, such as CCL19, CCL21, CXCL13, and lymphotoxin-α/β [14,15]. Furthermore, TLOs are sites of local activation of adaptive immunity, where locally antigen-activated B cells receive additional signals, clonally proliferate [16], and become a local source of donor-specific antibodies (DSAs) [12].

B cells are important mediators of TLO development due to their expression of lymphotoxin- α/β [17]. They can remain within allografts even after peripheral depletion of B cells using Rituximab [18]. B cell activating factor (BAFF) is an important survival factor for B cells and plasma cells [19]. In Ktx patients, elevated serum BAFF levels have also been associated with pre-sensitization, anti-HLA antibody formation, ABMR, and reduced graft survival [20,21]. We have previously demonstrated expression of BAFF in experimental rat kidney allografts [22], and also showed that anti-BAFF treatment interfered with humoral responses in Ktx using the same model [23]. However, a necessity of BAFF for TLO development has not previously been shown. Here, we tested the effects of an anti-BAFF antibody on intra-renal infiltrates of T and B cells, their microanatomical organization into TLOs, intra-renal expression of TLO-promoting factors and B cell differentiation factors, as part of local adaptive alloresponses in chronic rejection.

## 2. Results

### 2.1. Anti-BAFF Treatment Alters the T and B Cell Composition of Intra-Renal Infiltrates

**Figure 1:**
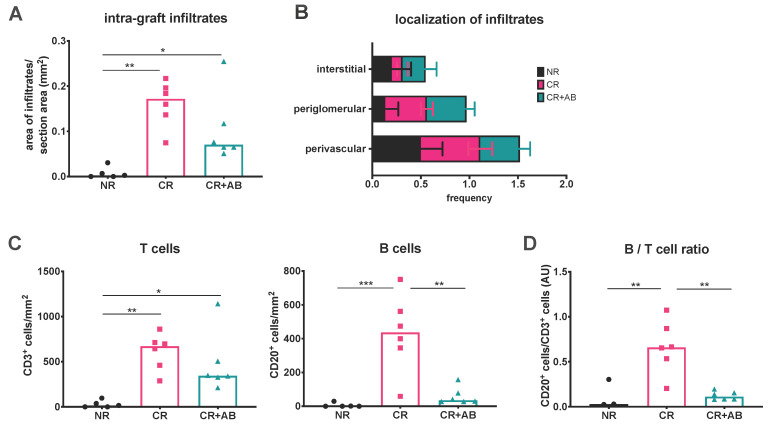
Intra-renal infiltrates, their microanatomical localization, and content of T and B lymphocytes. (**A**) shows intra-renal infiltrate expansion, which was measured using Histoquest software and was expressed as the cumulative area of infiltrates/area of the renal cortex. (**B**) shows the microanatomical localization of infiltrates, which was recorded as perivascular, periglomerular, or interstitial. (**C**) shows the intra-renal content of CD3^+^ T cells and CD20^+^ B cells, which was determined using Histoquest software after immunohistochemical staining and normalized to the area of renal cortex. (**D**) shows the ratio of intra-renal B/T cells in arbitrary units (AU). NR, no rejection (black); CR, chronic rejection (pink); CR + AB, chronic rejection and anti-BAFF antibody (green). Data is shown as individual data points per rat and group means. Statistical significance is shown as * *p* < 0.05, ** *p* < 0.01, and *** *p* < 0.001.

We first examined the overall area of intra-renal infiltrates in the different experimental groups using immunohistochemistry. Infiltrates were significantly more expansive in CR and CR + AB compared to NR (CR vs. NR: 0.16 ± 0.05 vs. 0.01 ± 0.01 mm^2^, *p* = 0.0012; CR + AB vs. NR: 0.10 ± 0.08 vs. 0.01 ± 0.01 mm^2^, *p* = 0.030) (Figure 1A). The expansion of intra-renal infiltrates appeared to be reduced in CR + AB compared to CR, but the difference was not significant. Analysis of the microanatomical localization of infiltrates showed that the majority of infiltrates were localized in the vicinity of arterioles (perivascular), followed by localization surrounding glomeruli (periglomerular) and few were located interstitially without apparent contact to arterioles or glomeruli (Figure 1B). We then assessed the number of T (CD3^+^) and B (CD20^+^) cells within kidney sections, and found that there were significantly more T cells in CR and CR + AB compared to NR (CR vs. NR: 610 ± 204 vs. 30 ± 40 cells/mm^2^, *p* = 0.0032; CR + AB vs. NR: 479 ± 338 vs. 30 ± 40 cells/mm^2^, *p* = 0.019), but CR and CR + AB did not differ significantly in intra-renal T cell content (Figure 1C). The number of B cells was also significantly elevated in CR compared to NR (CR vs. NR: 431 ± 232 vs. 6 ± 13 cells/mm^2^, *p* = 0.0006). Anti-BAFF treatment substantially reduced the number of intra-renal B cells (CR vs. CR + AB: 431 ± 232 vs. 60 ± 51 cells/mm^2^, *p* = 0.0013) (Figure 1C). Since T cells were non-significantly reduced in CR + AB compared to CR, we also assessed the ratio of B:T cells and found that this was elevated in CR compared to NR (0.67 ± 0.29 vs. 0.12 ± 0.16, *p* = 0.0067), and significantly reduced after anti-BAFF treatment (CR vs. CR + AB: 0.67 ± 0.29 vs. 0.12 ± 0.05, *p* = 0.0016) (Figure 1D).

### 2.2. Anti-BAFF Treatment Interfered with TLO Formation

**Figure 2:**
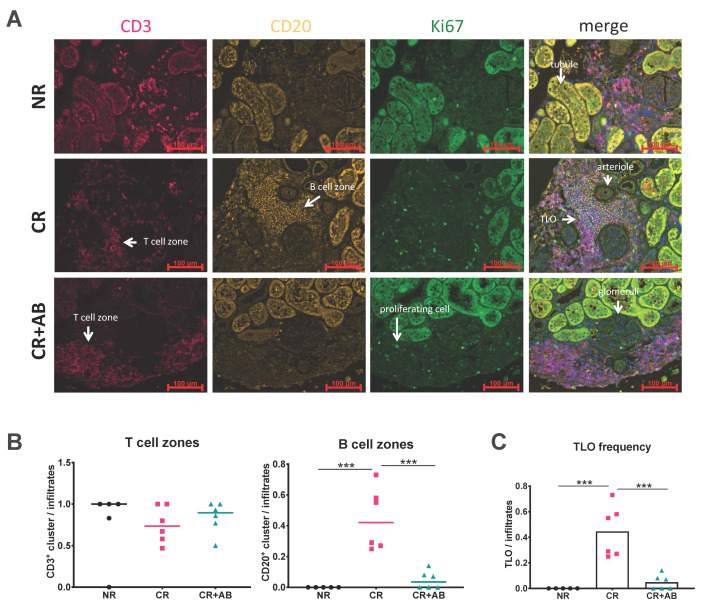
Effect of anti-BAFF treatment on intra-renal T and B cell zones and TLO formation. (**A**) shows representative allograft sections stained for CD3 (T cells, pink), CD20 (B cells, yellow), and Ki67 (proliferating cells, green) with distinct T and B cell zones. (**B**) shows the frequency of T cell (CD3^+^) and B cell (CD20^+^) zones, which were defined as dense clusters of predominantly one cell type. (**C**) shows the frequency of TLOs, which were defined as dense intra-renal infiltrates containing a T and a B cell zone. NR, no rejection (black); CR, chronic rejection (pink); CR + AB, chronic rejection and anti-BAFF antibody (green). Data is shown as group means and individual data points per rat. Statistical significance is shown as *** *p* < 0.001.

B cells and T cells can organize into distinct zones within infiltrates to form TLOs. We assessed the microanatomical organization of intra-renal T and B cells into T and B cell zones using immunofluorescence microscopy. Figure 2A shows representative images of staining of CD3^+^ T cells (red), CD20^+^ B cells (yellow), and Ki67^+^ proliferating cells (green). In NR, infiltrates were rare and small compared to the other groups. In CR, large infiltrates containing distinct B and T cell zones were found as shown in Figure 2A. Infiltrates after anti-BAFF treatment showed dense T cell zones but a lack of B cell zones. We determined the presence of T and B cell zones per infiltrate, and found that T cell zones were similarly frequent in all groups (Figure 2B), but the frequency of B cell zones within infiltrates was significantly higher in CR compared to NR (CR vs. NR: 0.44 ± 0.20 vs. 0.00 ± 0.00, *p* = 0.0001) but substantially lower with anti-BAFF treatment (CR vs. CR + AB: 0.44 ± 0.20 vs. 0.05 ± 0.06, *p* = 0.0002) (Figure 2B). TLOs were defined by the presence of T and B cell zones, and were absent in NR but significantly elevated in CR (CR vs. NR: 0.44 ± 0.20 vs. 0.00 ± 0.00, *p* = 0.0001) (Figure 2C). However, the frequency of TLO was significantly diminished after anti-BAFF treatment (CR vs. CR + AB: 0.44 ± 0.20 vs. 0.048 ± 0.06, *p* = 0.0002) (Figure 2C).

### 2.3. Proliferation of Intra-Renal T and B Cells Was Not Altered by Anti-BAFF Treatment

**Figure 3:**
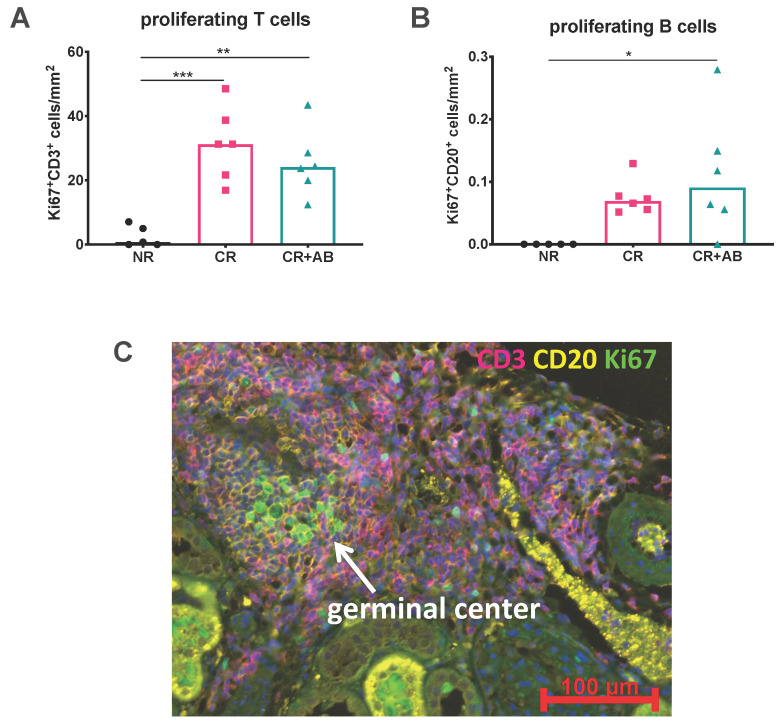
Intra-renal T and B cell proliferation and germinal center (GC) formation within TLOs. (**A**) shows the intra-renal content of proliferating Ki67^+^CD3^+^ T cells and (**B**) proliferating Ki67^+^CD20^+^ B cells, which were quantified using Histoquest software after immunohistochemical staining and normalized to the area of renal cortex. (**C**) shows a TLO with GC formation, which was defined as a dense cluster of Ki67^+^ proliferating cells (green) within a CD20^+^ B cell zone (yellow); the kidney section was from the CR group. NR, no rejection (black); CR, chronic rejection (pink); CR + AB, chronic rejection and anti-BAFF antibody (green). Data is shown as group means and individual data points per rat. Statistical significance is shown as * *p* < 0.05, ** *p* < 0.01, and *** *p* < 0.001.

Since the number of B cells was significantly reduced within kidney allografts by anti-BAFF treatment, we were interested in whether anti-BAFF treatment had affected the local proliferation of intra-renal lymphocytes. We therefore analyzed the absolute numbers of proliferating T and B cells by co-staining for the proliferation marker Ki67^+^. We found that proliferation of T cells was significantly increased in CR and CR + AB compared to NR (CR vs. NR: 31 ± 11 vs. 3 ± 3, *p* = 0.0005; CR + AB vs. NR: 25 ± 10 vs. 3 ± 3, *p* = 0.0034). In B cells, only CR + AB showed significantly increased proliferation compared to NR, while elevation of B cell proliferation in CR was not significant compared to NR (CR + AB vs. NR: 0.11 ± 0.04 vs. 0 ± 00, *p* = 0.02; CR vs. NR: 0.08 ± 0.03 vs. 0 ± 00, *p* = 0.137). However, anti-BAFF treatment did not change the number of proliferating T cells (Figure 3A) or B cells (Figure 3B).

Antigen-activated B cells may clonally proliferate in germinal centers (GCs) when given appropriate T helper cell signals. We analyzed infiltrates for the presence of GCs within TLOs and found that GCs were rare but could be detected in CR, as shown in Figure 3C.

### 2.4. Effect of Anti-BAFF Treatment on Intra-Renal Plasma Cells

**Figure 4:**
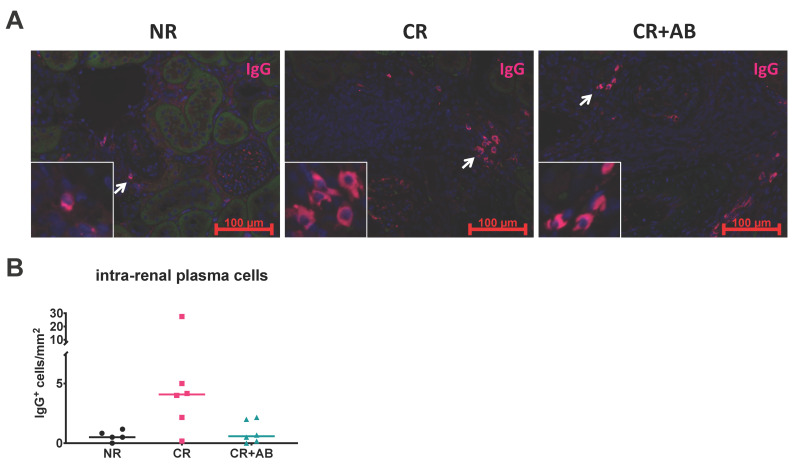
Effect of anti-BAFF treatment on intra-renal plasma cells. (**A**) shows immunofluorescence staining of IgG^+^ plasma cells typically arranged in small nests within allografts. (**B**) shows the intra-renal content of IgG^+^ plasma cells, which was determined by blinded manual counting of IgG^+^ cells and normalized to the renal cortex area. NR, no rejection (black); CR, chronic rejection (pink); CR + AB, chronic rejection and anti-BAFF antibody (green). Data is shown as group means and individual data points per rat.

Plasma cells are antibody-producing cells derived from B cells and have been found within kidney allograft infiltrates [24]. We therefore determined the amount of intra-renal plasma cells by staining for IgG^+^ elliptical cells, as previously described [23]. Plasma cells were present in allografts from all groups and appeared increased in CR compared to NR and CR + AB, but the difference was not statistically significant (Figure 4).

### 2.5. Anti-BAFF Treatment Regulated Expression of TLO-Promoting Factors and B Cell Differentiation Markers within Allografts

**Figure 5:**
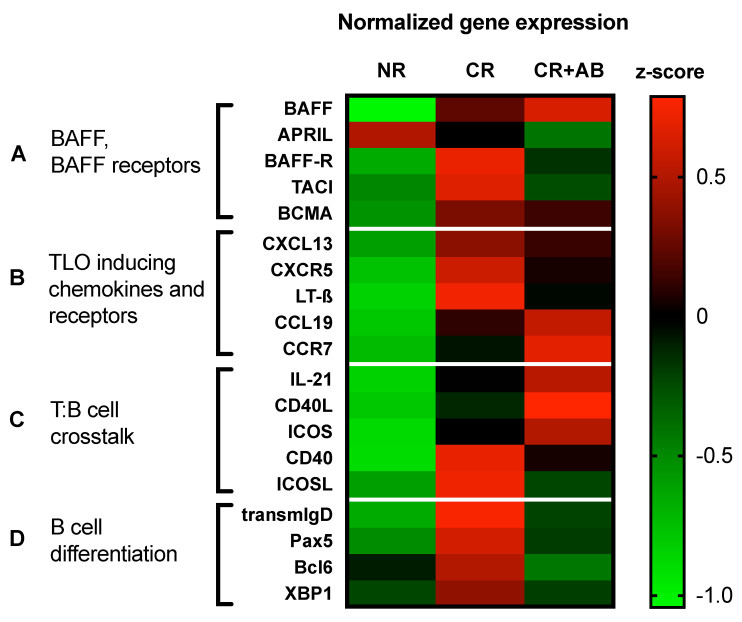
Effect of anti-BAFF treatment on intra-renal gene expression in chronic rejection. A heat-map was used to visualize relative mRNA expression of genes between groups. (**A**) BAFF, APRIL, and their receptors; (**B**) TLO-inducing chemokines and cytokines and receptors; (**C**) molecules of T:B cell crosstalk; (**D**) markers of B cell differentiation. MRNA expression of target genes was normalized to the house-keeper hypoxanthine-guanine-phosphoribosyl-transferase (HPRT,). The z-score was calculated from delta CT values per sample using the mean of all samples. Average z-scores from each group are shown using the indicated color scale for up- or downregulation.

Since BAFF was required for TLO formation, we were interested in the expression of TLO-promoting factors and B cell activation and differentiation molecules. To this end, we used a heat-map to visualize normalized gene expression (Figure 5). We analyzed the intra-renal mRNA expression of BAFF, its homolog APRIL (a proliferation-inducing ligand), and their receptors, BAFF-R (BAFF receptor), TACI (transmembrane activator and calcium modulator and cyclophilin ligand interactor), and BCMA (B cell maturation antigen). We found that BAFF and its receptors were more strongly expressed in CR than NR, but anti-BAFF treatment led to decreased expression of BAFF-R, TACI, and to a lesser degree BCMA (Figure 5A).

TLO formation is driven by lymphoid chemokines and cytokines. We therefore measured intra-renal mRNA expression of the B cell chemokine CXCL13 and its receptor CXCR5, as well as lymphotoxin-β and the T cell chemokine CCL19 and its receptor CCR7. We found that expression of B cell chemokine CXCL13 and its receptors CXCR5, as well as lymphotoxin-β, was elevated in CR but reduced after anti-BAFF, while expression of T cell chemokine CCL19 and its receptor CCR7 was increased in CR + AB (Figure 5B).

We found evidence of germinal center formation in CR, but anti-BAFF treatment did not affect the number of proliferating B cells within allografts. We therefore analyzed the expression of molecules, which drive B cell proliferation. We found that expression of interleukin (IL)-21, an important T cell cytokine, which stimulates B cell proliferation, was elevated in CR + AB compared to CR (Figure 5C). Similarly, the expression of T cell costimulatory molecules, CD40L (CD40 ligand) and ICOS (inducible T cell costimulator), was higher in CR + AB compared to CR, while expression of corresponding B cell ligands CD40 and ICOS ligand was lower in CR + AB compared to CR (Figure 5C).

Finally, BAFF is a survival factor for B cells at different differentiation stages. Therefore, we assessed the intra-renal expression of markers of B cell differentiation. Transmembrane IgD (transmemIgD) is expressed by immature and naïve B cells and its expression was elevated in CR and substantially reduced in CR + AB (Figure 5D). Pax5 (paired box protein 5), a pan-B cell lineage factor, was also elevated in CR and reduced in CR + AB (Figure 5D). Bcl-6 (B cell lymphoma 6) is expressed by germinal center B cells after activation in B cell follicles and was elevated in CR but lower in CR + AB (Figure 5D). Finally, XBP-1 (X-box binding protein 1) is a transcription factor for differentiating plasma cells and its intra-renal expression was elevated in CR compared to NR, and anti-BAFF treatment reduced its expression compared to CR (Figure 5D). Overall, early B cell differentiation markers appeared to be increased in CR and more strongly reduced by anti-BAFF treatment than markers of later differentiation stages (Figure 5D).

Since we saw no significant difference in the amount of T cells present within allografts (Figure 1) or their proliferation rate (Figure 3) with or without anti-BAFF treatment, we analyzed the expression of molecules associated with T cell function by qPCR. We found no difference in the expression of interleukin-2, interferon-γ, interleukin-21, TGF-β, granzyme B, perforin, or foxp3 with or without anti-BAFF treatment (data not shown).

## 3. Discussion

TLOs are sites of adaptive alloimmune activation and have been detected in failed kidney allografts with chronic rejection [11]. We tested if BAFF is required for TLO formation in a model of chronic kidney allograft rejection in rats. Our results showed that intra-renal infiltrates were more frequent in chronic rejection than in non-rejecting allografts and included TLOs with distinct T and B cell zones. Furthermore, we observed B cell germinal center formation within TLOs. Anti-BAFF treatment diminished the number of intra-renal B cells and effectively blocked the formation of B cell zones and TLOs. Gene expression analysis showed that intra-renal expression of BAFF receptors and TLO-promoting factors was elevated in CR but diminished by anti-BAFF treatment. We found that B cells in early differentiation stages were particularly sensitive to anti-BAFF treatment. In summary, we showed that TLOs harbor local activation of alloresponses and that BAFF is required for TLO formation in chronic rejection.

In fully immunosuppressed non-rejecting allografts, intra-renal infiltrates were virtually absent; however, there was significant expansion of infiltrates within allografts in chronic rejection. Intra-renal infiltrates were most frequently found in the vicinity of arterioles, suggesting leukopedesis across existing blood vessels, rather than sprouting of high endothelial venules, which have been described in TLOs [14]. Anti-BAFF treatment did not affect the expansion of intra-renal infiltrates; it did, however, lead to a significant reduction in the number of intra-renal B cells and a lower B/T cell ratio.

Detailed analysis of the microanatomical organization of intra-renal T and B cells showed that a large portion of infiltrates consisted of distinct T and B cell zones forming lymphoid follicle-like structures or TLOs. Staining of the proliferation marker Ki67 revealed that germinal centers, or areas of clonal proliferation of antigen-specific B cells, could form within these TLOs in our model. Germinal centers are highly advanced structures, which arise from coordinated T:B cell crosstalk [25]. It is within germinal centers that B cells get fully activated, go through somatic hypermutation, get positively selected for the highest antibody affinity for alloantigens, and differentiate into antibody-secreting plasma cells and memory B cells [26]. Cheng et al. have previously shown that TLOs can harbor such diversification and clonal expansion of alloimmune responses in allografts [16]. Our results showed that anti-BAFF treatment can effectively prevent the formation of intra-renal TLOs, since B cell zones were mostly absent in anti-BAFF-treated rats. Although TLO formation has been observed in cardiac transplant models in mice [27], this is the first kidney transplant model showing TLO formation in chronic rejection. As such, the findings from our study may provide a basis for further investigation into the role of TLOs in kidney transplant rejection.

We were interested in the mechanism by which anti-BAFF treatment prevented TLO formation in allografts. There are three known receptors for BAFF and its homolog APRIL: BAFF-R, TACI, and BCMA, as reviewed by Parsons [28]. We found that the intra-renal expression of BAFF receptors was significantly lower after anti-BAFF treatment, which illustrated the dependency of BAFF receptor-expressing cells on the presence of BAFF as a survival factor. Whether there was a shortage of infiltrating B cells into allografts or intra-renal B cells locally succumbed to cell death in the absence of BAFF could not be determined by our experiments. CXCL13, a B cell chemokine that promotes TLO formation, was highly expressed during chronic rejection, but expression appeared lower after anti-BAFF treatment. The expression of CXCR5, the receptor for CXCL13, was also lower after anti-BAFF treatment. Furthermore, B cells are potent producers of lymphotoxin-β, an important factor for secondary and tertiary lymphoid organ formation [29,30]. The expression of lymphotoxin-β was elevated in chronic rejection but substantially diminished after anti-BAFF antibody. The regulation of these factors may have played in important role in preventing the development of TLO in anti-BAFF-treated rats in our model.

Intra-renal mRNA expression of B cell differentiation markers showed that anti-BAFF treatment strongly affected B cells at early differentiation stages and to a lesser extent reduced expression of advanced differentiation markers. Transmembrane IgD is expressed by immature transitional and naïve B cells. We found elevated transmembrane IgD expression in chronic rejection, reflecting the infiltration of these cells into allografts. Interestingly, expression of intra-renal transmembrane IgD was substantially reduced by anti-BAFF treatment. We also found increased expression of Pax5, a pan-B cell lineage transcription factor, within allografts with chronic rejection but a significant reduction of its expression after anti-BAFF treatment. Interestingly, the transcription factor Bcl-6 was also upregulated during chronic rejection but downregulated by anti-BAFF treatment. Bcl-6 is expressed in lymphoid follicles by germinal center B cells and T follicular helper cells, which provide essential activation signals to B cells, as reviewed by Vinuesa [31]. Bcl-6 is therefore a necessary factor for full activation of humoral alloresponses. Decreased Bcl-6 expression in rats treated with anti-BAFF antibody could reflect decreased humoral immune activation in allografts due to a lack of TLOs. Finally, expression of XBP-1, a transcription factor expressed during plasma cell differentiation [32], was elevated in chronic rejection. XBP-1 expression was also reduced in anti-BAFF, but the difference was much less pronounced compared with factors of early B cell differentiation. We investigated the presence of plasma cells within allografts in our model. Although the exact effector function of intra-renal plasma cells and their role in allograft rejection is not fully understood, intra-renal plasma cells have been associated with chronic allograft rejection and poor prognosis [24,33,34,35,36]. Although we have previously shown that anti-BAFF therapy reduced the number of splenic plasma cells [23], the difference in intra-renal plasma cells between groups was not statistically significant in this study. In summary, we found that intra-renal B cells during early differentiation stages were particularly sensitive to anti-BAFF treatment.

Signals required for B cell proliferation include antigen-activation, T cell help, and cytokines, but not BAFF, which was reflected by our data showing that B cell proliferation was not altered by anti-BAFF treatment. Analysis of B cell differentiation factors showed that early antigen-naïve B cells were particularly reduced by anti-BAFF treatment, suggesting that the remaining B cells were already antigen-activated. Proliferation of antigen-activated B cells depends on the expression of important signals by T helper cells, namely CD40L and IL-21. In line with this, we found that the relative expression of CD40L and IL-21 was not decreased by anti-BAFF treatment, demonstrating a potentially important gap in the mechanism of BAFF. This observation may point towards a shortcoming of BAFF-targeted therapy and raise interest in combining targets for therapy.

We demonstrated that TLOs are sites of humoral immune activation within allografts during chronic rejection, and that anti-BAFF treatment can hinder the formation of TLO in allografts. Belimumab^®^, a monoclonal anti-BAFF antibody, has been approved for treatment of lupus erythematodes and has been explored as an immunosuppressive agent in kidney transplant patients [37]. Our study shows that TLO formation may be prevented using an anti-BAFF antibody with a potential benefit to kidney transplant patients. We previously reported that anti-BAFF treatment interfered with systemic humoral responses and reduced formation of DSA of certain IgG subclasses in a Ktx model, but we found no direct evidence for any specific alteration in T cell function and it did not significantly affect allograft rejection according to Banff or allograft function [23]. A limitation of our study is the duration of our experiment. Extending experiments beyond d56 post-transplant may provide insight into the long-term effects of anti-BAFF treatment on cellular responses within allografts. To assess the long-term benefits of such an intervention, further investigation is needed.

## 4. Materials and Methods

### 4.1. Experimental Kidney Transplantation

Animal experiments were approved by the local inspecting authorities (Regierung von Unterfranken, No. 55.2-2532-2-47, 30-06-2015) and performed according to German animal protection laws and NIH’s laboratory animal care principles. In brief, a previously described MHC-mismatched rat kidney transplantation model was used [22,23], in which male Brown Norway rats (BN) served as donors and male Lewis rats (LEW) as recipients (Charles River Laboratories, Sulzfeld, Germany, 200–250 g). Kidney transplantation was performed orthotopically as previously described [38].

Rats were either treated with daily cyclosporine A (CsA) at 10 mg/kg body weight (Neoral, Novartis, Basel, Switzerland), administered once daily by gavage (no rejection group, NR), or they received CsA 5 mg/kg daily until d6, then on every 2nd day to induce DSA and chronic rejection (CR). In addition, one group received CsA 5 mg/kg daily until d6, then on every 2nd day, and in addition a monoclonal anti-BAFF antibody (GSK, Hamburg, Germany) was injected in the peritoneal cavity on d3, d17, d31, and d45 after Ktx (CR + AB), as previously described [23]. Experimental groups are shown in Table 1 below. Rats were sacrificed on d56 after Ktx and allografts were harvested.

### 4.2. Histology, Immunohistochemistry, and Immunofluorescence

Immunohistochemistry was performed on 3-μm formalin-fixed paraffin-embedded sections as previously described [39]. T cells were stained with rabbit anti-rat CD3 antibody (Abcam, ab5690, Cambridge, UK) and donkey anti-rabbit-Cy5 (Dianova, 711-175-152, Hamburg, Germany). B cells were stained with mouse anti-rat CD20 antibody (Santa Cruz, sc-393894, Heidelberg, Germany), goat anti-mouse-biotin (Thermofisher 31804), and Strep-Cy3 (Dianova, 016-160-084, Hamburg, Germany). Proliferation was stained using anti-Ki67-FITC (ebioscience, 11-5698), and plasma cells were stained using anti-rat-IgG-AlexaFluor647 (Thermofischer A21472, Waltham, MA, USA). Images were taken using a Zeiss observer Z.1 Fluorescence microscope at 20× magnification. Digital images from 10 high power fields (HPFs) per specimen were examined as previously described [39]. Using Histoquest^®^ software (TissueGnostics GmbH, Vienna, Austria), the number of CD20^+^, CD3^+^, Ki67^+^CD20^+^, and Ki67^+^CD3^+^ cells were quantified within a defined section area. Intra-renal B cell (CD20^+^) and T cell (CD3^+^) zones were defined as dense clusters of predominantly one cell type (min. 20 cells/0.004 mm^2^). TLO were defined as dense intra-renal infiltrates containing a T and B cell zone. Germinal centers (GCs) were defined as dense clusters of Ki67^+^ proliferating cells within a B cell zone. The presence of T and B cell zones and GC was determined per infiltrate in a blinded manner. Intra-renal IgG^+^ plasma cells, identified by strong intracellular IgG-positivity and typical cell shape, were counted manually in a blinded manner.

### 4.3. Real-Time PCR

Frozen tissue sections were homogenized using a RNeasy MiniKit^®^ (cat. 74106, Qiagen, Hilden, Germany). Total RNA was extracted, and genomic DNA digested. Total RNA was reverse transcribed into cDNA. RT-PCR was performed on a ViiA7 detection system (Applied Biosystems, Darmstadt, Germany) using a QuantiTect SYBR Green PCR Kit (Qiagen, Hilden, Germany). The sequences of the primers are listed in Table S1). Copy numbers of target genes were normalized to the house-keeper gene hypoxanthine-guanine-phosphoribosyl-transferase (HPRT) and delta CT values were calculated. The z-score was calculated from delta CT values for each sample and target gene using the formula z = (χ-μ)/σ. Mean z-scores from each experimental group are shown in a heat map using a color scale for up- or downregulation of gene expression.

### 4.4. Statistical Analysis

Values are provided as individual data points and mean and SD. Statistical significance was calculated using Graphpad Prism software (Version 8.0, San Diego, CA, USA) using ANOVA; *p* < 0.05 was considered to be statistically significant.

## Figures and Tables

**Table 1:** Experimental groups.

Group	Abbreviation	n=
Ktx CsA 10 mg/kg/d d56 (no rejection)	NR	5
Ktx CsA 5 mg/kg/2^nd^ d d56 (chronic rejection)	CR	6
Ktx CsA 5 mg/kg/2nd d d56 + anti-BAFF antibody (chronic rejection + anti-BAFF antibody)	CR + AB	6

## Supplementary Materials

The following are available online at https://www.mdpi.com/1422-0067/21/21/8045/s1. Table S1: Primer sequences.

## Abbreviations

AB	antibody
ABMR	antibody-mediated rejection
APRIL	a proliferation inducing ligand
BAFF	B cell activating factor
BAFF-R	BAFF receptor
Bcl-6	B cell lymphoma 6
BCMA	B cell maturation antigen
BN	Brown Norway rat
CD	cluster of differentiation
CD40L	CD40 ligand
CR	chronic rejection
CR + AB	chronic rejection + anti-BAFF antibody
CsA	cyclosporine A
HPRT	hypoxanthine-guanine-phosphoribosyl-transferase
GC	germinal center
ICOS	inducible T cell costimulator
ICOSL	ICOS ligand
IFTA	interstitial fibrosis and tubular atrophy
Ig	immunoglobulin
IL	interleukin
Ktx	kidney transplantation
LEW	Lewis rat
MHC	major histocompatibility complex
NR	no rejection
Pax5	paired box protein 5
SLE	systemic lupus erythematosus
TACI	transmembrane activator and calcium modulator and cyclophilin ligand interactor
TLO	tertiary lymphoid organs
XBP-1	X-box binding protein 1
