# Supplementary material for: B Cell Activating Factor (BAFF) Is Required for the Development of Intra-Renal Tertiary Lymphoid Organs in Experimental Kidney Transplantation in Rats
Source: Int J Mol Sci. 2020 Oct 28;21(21):8045. doi: 10.3390/ijms21218045 (PMC7662293; doi:10.3390/ijms21218045)
Supplement: Supplementary file 1 [file ijms-21-08045-s001.pdf]

Table S1

Primer sequences:

rAPRIL forw (29) (5'- ATC CTG ACC GTG CCT ACA AC -3')

rAPRIL rev (30) (5'- TCA CAA ACC CCA GGA ATG TT -3')

rBAFF_for: (5'- GAC CGG AGG AAA CAG AAC AA -3')

rBAFF_rev: (5'- TGC AAT CAG CTG CAG ACA GT -3')

rBAFF-R forw2 (73) (5'-GTG GGT CTG GTG AGT CTG GT-3')
rBAFF-R rev2 (74) (5'-CAT TTT CCA GGG ACT CTT GC-3')

rBCL6 forw1 (5‘-CTG AGG GAA GGC AAC ATC AT-3’)

rBCL6 rev1 (5‘-CGG CTG TTC AGG AAC TCT TC-3’)

rBCMA forw (5'-CGT CTG TTT GGC ACT TTT CA-3')
rBCMA rev (5'-CAC CAG CCC TGC TCT TAG TC-3')

rCCL19 forw (99) (5'- AGA CTG CTG CCT GTC TGT GA -3')

rCCL19 rev (100) (5'- GCT GGT AGC CCC TTA GTG TG -3')

rCCR7 forw: 5'-GGT CAT TTT CCA GGT GTG CT-3

rCCR7 rev: 5'-AGT TCC GCA CAT CCT TCT TG-3

rCD40_forw2 (5'-GGA CAG TGT GTT ACG TGC AG-3')

rCD40_rev2 (5'-GGT TGG CAT TGG GTC TTC TC-3')

rCD40Lig_forw2 (5‘-AGA TGA TTG GGT CGG TGC TT-3’)

rCD40Lig_rev2 (5‘-CCC TTC TCC TTT GTT GCA CC-3’)

rCXCL13 forw: 5‘- GCA AAA ATC AGG CTT CCA GA -3‘
rCXCL13 rev: 5‘- GGG TCA CAG TGC AAA GGA AT -3‘

rCXCR5forw2: 5’-TCC CGG TTT CTC TAC CAC AC-3’

rCXCR5rev2: 5'-CCA GCA GAG AAG GAA GAT GC-3'

rHPRT forw (5'-CTTTGGTCAAGCAGTACA GCC-3')

rHPRT rev (5'-TCCGCTGATGACACAAACATGA-3')

rICOS_forw2 (5‘-AAT CCC AGC TTT GTT GCC AG-3’)

rICOS_rev2 (5‘-TCG TGC ACA CTG GAT CTG TA-3’)

rICOSLig_forw (5'-TGG ATC AAC AGG ACG GAC AA-3')

rICOSLig_rev (5'-GGA TTT CCT GTG GCC TCT CT-3')

rIgD-trmem forw2 (5'- GCT CTA CAG TGG CTT TGT CA -3')

rIgD-trmem rev2 (5'- CTG TGG GCT TCA TGT TGA GG -3')

rIL-6_forw2: 5‘- AGC CAG AGT CAT TCA -3
rIL-6_rev2: 5‘- AGA GCA TTG CAA GTT GGG GT -3

rIL-10_forw2: 5‘- GCT GGA CAA CAT ACT GCT GA -3‘
rIL-10_rev2: 5‘- GGG CAT CAC TTC TAC CAG GT -3‘

rIL-21_BT forw2 (5’-CGA AGC TTT TGC CTG TTT TC-3’)

rIL-21_BT rev2 (5’-CAA ATC ACA GGA AGG GCA TT-3’)

rLTB forw: 5'-TAT CAC TGT CCT GGC TGT GC-3'

rLTB rev: 5'-GAG ATG CAC GAG GGT TTG TT-3'

rTGF-b_forw2 (5’-CAT GCC AAC TTC TGT CTG GG-3’)

rTGF-b_rev2 (5’-CGG GTT GTG TTG GTT GTA G-3’)

rTACI forw1 (5'- GGC CGG ATA ACT TAG GAA GG -3')

rTACI rev1 (5'- TGG GAA GTG GCT CTC CTC TA -3')

rPAX-5_for (5'- AGA TGC GGG GAG ATC TGT TC -3')

rPAX-5_rev (5'- CCT GTG ACA ATG GGG TAG GA -3')

rXBP-1_for (5'- GCC CTG GTT ACT GAA GAG GT -3')

rXBP-1_ rev (5'- GAT GTT CTG GGG AGG TGA CA -3')
